# Changes in breast cancer treatment during the COVID-19 pandemic: a Dutch population-based study

**DOI:** 10.1007/s10549-022-06732-y

**Published:** 2022-11-05

**Authors:** Anouk H. Eijkelboom, Linda de Munck, C. Willemien Menke-van der Houven van Oordt, Mireille J. M. Broeders, Desiree H. J. G. van den Bongard, Luc J. A. Strobbe, Marc A. M. Mureau, Marc B. I. Lobbes, Pieter J. Westenend, Linetta B. Koppert, Agnes Jager, Ester J. M. Siemerink, Jelle Wesseling, Helena M. Verkooijen, Marie-Jeanne T. F. D. Vrancken Peeters, Marjolein L. Smidt, Vivianne C. G. Tjan-Heijnen, Sabine Siesling, The NABON-COVID-19 Consortium, The COVID and Cancer Care-NL Consortium, J. C. van Hoeve, M. A. W. Merkx, N. J. de Wit, I. Dingemans, I. D. Nagtegaal

**Affiliations:** 1 Department of Research and Development, Netherlands Comprehensive Cancer Organisation (IKNL), Utrecht, The Netherlands; 2 Department of Health Technology and Services Research, University of Twente, Drienerlolaan 5, 7522 NB Enschede, The Netherlands; 3 Department of Medical Oncology, Cancer Center Amsterdam, Amsterdam UMC Location VUMC, Amsterdam, The Netherlands; 4 Department for Health Evidence, Radboud University Medical Center, Nijmegen, The Netherlands; 5 Dutch Expert Centre for Screening, Nijmegen, The Netherlands; 6 Department of Radiation Oncology, Amsterdam UMC, Amsterdam, The Netherlands; 7 Department of Surgical Oncology, Canisius Wilhelmina Hospital, Nijmegen, The Netherlands; 8 Department of Plastic and Reconstructive Surgery, Erasmus MC Cancer Institute, Erasmus University Medical Center, Rotterdam, The Netherlands; 9 Department of Medical Imaging, Zuyderland Medical Center Sittard-Geleen, Geleen, The Netherlands; 10 Department of Radiology and Nuclear Medicine, Maastricht University Medical Center, Maastricht, The Netherlands; 11 School for Oncology and Developmental Biology (GROW), Maastricht University, Maastricht, The Netherlands; 12 Laboratory of Pathology, Dordrecht, The Netherlands; 13 Department of Surgery, Erasmus University Medical Center, Rotterdam, The Netherlands; 14 Department of Medical Oncology, Erasmus MC Cancer Institute, Erasmus University Medical Center, Rotterdam, The Netherlands; 15 Department of Internal Medicine, Ziekenhuis Groep Twente, Hengelo, The Netherlands; 16 Division of Diagnostic Oncology and Molecular Pathology, Netherlands Cancer Institute – Antoni van Leeuwenhoek Hospital, Amsterdam, The Netherlands; 17 Department of Pathology, Leiden University Medical Center, Leiden, The Netherlands; 18 Division of Imaging and Oncology, University Medical Centre Utrecht, Utrecht, The Netherlands; 19 Department of Surgery, Netherlands Cancer Institute – Antoni van Leeuwenhoek hospital, Amsterdam, The Netherlands; 20 Department of Surgery, Amsterdam UMC, Amsterdam, The Netherlands; 21 Department of Surgery, Maastricht University Medical Centre, Maastricht, The Netherlands; 22 Department of Medical Oncology, School for Oncology and Developmental Biology (GROW), Maastricht University Medical Centre, Maastricht, The Netherlands; 23 Department of Oral and Maxillofacial Surgery, Radboud University Nijmegen Medical Centre, Nijmegen, The Netherlands; 24 Department of General Practice, Julius Center for Health Sciences and Primary Care, University Medical Center Utrecht (UMCU), Utrecht University, Utrecht, The Netherlands; 25 Dutch Federation of Cancer Patient Organisations (NFK), Utrecht, The Netherlands; 26 Department of Pathology, Radboud University Nijmegen Medical Centre, Nijmegen, The Netherlands

**Keywords:** COVID-19, Breast cancer, Treatment, Breast cancer care

## Abstract

**Purpose:**

We aimed to compare (1) treatments and time intervals between treatments of breast cancer patients diagnosed during and before the COVID-19 pandemic, and (2) the number of treatments started during and before the pandemic.

**Methods:**

Women were selected from the Netherlands Cancer Registry. For aim one, odds ratios (OR) and 95% confidence intervals (95%CI) were calculated to compare the treatment of women diagnosed within four periods of 2020: pre-COVID (weeks 1–8), transition (weeks 9–12), lockdown (weeks 13–17), and care restart (weeks 18–26), with data from 2018/2019 as reference. Wilcoxon rank-sums test was used to compare treatment intervals, using a two-sided *p*-value < 0.05. For aim two, number of treatments started per week in 2020 was compared with 2018/2019.

**Results:**

We selected 34,097 women for aim one. Compared to 2018/2019, neo-adjuvant chemotherapy was less likely for stage I (OR 0.24, 95%CI 0.11–0.53), stage II (OR 0.63, 95%CI 0.47–0.86), and hormone receptor+/HER2− tumors (OR 0.55, 95%CI 0.41–0.75) diagnosed during transition. Time between diagnosis and first treatment decreased for patients diagnosed during lockdown with a stage I (*p* < 0.01), II (*p* < 0.01) or III tumor (*p* = 0.01). We selected 30,002 women for aim two. The number of neo-adjuvant endocrine therapies and surgeries starting in week 14, 2020, increased by 339% and 18%, respectively. The number of adjuvant chemotherapies decreased by 42% in week 15 and increased by 44% in week 22.

**Conclusion:**

The pandemic and subsequently altered treatment recommendations affected multiple aspects of the breast cancer treatment strategy and the number of treatments started per week.

**Supplementary Information:**

The online version contains supplementary material available at 10.1007/s10549-022-06732-y.

## Introduction

**Fig. 1:**
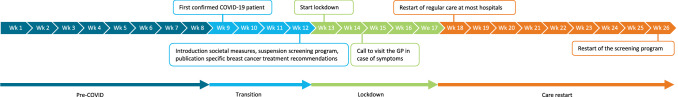
Dutch COVID-19 timeline of 2020, *GP* general practitioner

In December, 2019, the first infections with SARS-COV-2, the virus causing COVID-19, were confirmed in Wuhan, China [1]. Thereafter, the virus quickly spread around the world. In the Netherlands, the first COVID-19 case was confirmed on February 27, 2020 (week 9) (Fig. 1). After week 9, the number of hospitalized COVID-19 patients quickly increased, putting an enormous pressure on healthcare, including breast cancer care. To mitigate spreading of the virus societal measures were taken in week 12. These societal measures, together with the increased pressure on healthcare, forced the national breast cancer screening program to a halt in week 12. In week 13 the country went into lockdown. The suspension of the screening program, together with the reluctance of patients to visit the general practitioner (GP), as well as the reduced capacity at the GP led to a drop in the incidence of cancer diagnoses [2]. In weeks 14 and 15, there was therefore a national call to visit the GP in case of symptoms. From week 14 onwards there was a slow decrease in the number of hospitalized COVID-19 patients. In most hospitals, this allowed resumption of regular care from week 18 onwards [3]. The breast cancer screening program was resumed at a 40% capacity in week 26, and this capacity slowly increased in the weeks thereafter.

At the start of the pandemic multiple international guidelines, as well as Dutch guidelines, were introduced to ensure safe and effective oncological care for all breast cancer patients during the pandemic (Table 1) [4–9]. These COVID-19 induced recommendations discouraged the use of neo-adjuvant chemotherapy in patients planned for surgery [4], while they encouraged the use of neo-adjuvant endocrine therapy to postpone surgery [4–7]. The lowest priority was given to the surgical management of low-grade DCIS [5–7]. Recommendations discouraged the use of immediate breast reconstructions (IBR) with autologous tissue [4] or IBR altogether [6, 8]. Finally, adjuvant radiotherapy could be used before, instead of after, chemotherapy in carefully selected patients [6]. It was expected that hospitals used these COVID-19 induced recommendations, together with their expertise, to change the treatment of breast cancer patients during the pandemic if needed.

**Table 1:** Overview of the recommendations on how to prioritize and adapt treatment for breast cancer patients during the COVID-19 pandemic

Recommendations	Source
Neo-adjuvant chemotherapy should only be used for inoperable patients	Associations of breast surgery [4]
Neo-adjuvant endocrine therapy can be used to postpone surgery	Association of breast surgery [4], European Association of Medical Oncology (ESMO) [5], COVID-19 pandemic breast cancer consortium [6], Dutch Society of Medical Oncology (NVMO), Dutch Society of Surgical Oncology (NVCO) [7]
Lowest priority should be given to the surgery of low-grade DCIS	European Association of Medical Oncology (ESMO) [5], COVID-19 pandemic breast cancer consortium [6], Dutch Society of Medical Oncology (NVMO), Dutch Society of Surgical Oncology (NVCO) [7]
Immediate breast reconstructions should be avoided	Associations of Breast Surgery [4]
Immediate breast reconstruction with autologous tissue has the lowest priority	COVID-19 pandemic breast cancer consortium [6], American Society of Plastic Surgeons [8]
Chemotherapy can be used after radiotherapy in carefully selected patients	COVID-19 pandemic breast cancer consortium [6]

Previous multi-centered studies investigating the effect of the COVID-19 pandemic, and the subsequently altered recommendations, on the treatment received by breast cancer patients were relatively small, including between 217 and 3776 patient [10–14], or only looked at the effect of the pandemic on the initial treatment of breast cancer patients [15]. Studies investigating the effect of the pandemic on breast cancer treatment are a crucial first step to enable accurate evaluation of the consequences of changes in the treatment strategies on risk of recurrence and survival of breast cancer patients. This might provide valuable insights into how treatment can be improved, both now and during future situations with limited resources. Therefore, the current study aimed to compare 1) the treatments and the time intervals between treatments of patients diagnosed in weeks 1–26 of 2020 and patients diagnosed in 2018/2019, and 2) the number of treatments started per week during weeks 2–26 of 2020 and weeks 2–26 of 2018/2019.

## Methods

Women were selected from the NCR. The NCR has records of all newly diagnosed malignancies notified through the Nationwide Histopathology and Cytopathology Data Network and Archive (PALGA) since 1989. Women were excluded if they were younger than 18 years, had a history of breast cancer or a synchronous tumor (detected within 91 days), or did not receive surgery in case of an invasive tumor.

Stage (DCIS and stage I, II, III, IV) was defined according to the TNM staging system [16]. Hormone receptor status (HR) and human epidermal growth factor receptor 2 (HER2) status were combined in the variable ‘tumor subtype’ (HR+/HER2+, HR+/HER2−, HR−/HER2+, HR−/HER2−).

### Statistical analysis

#### Aim 1: impact on the breast cancer treatment strategy

**Fig. 2:**
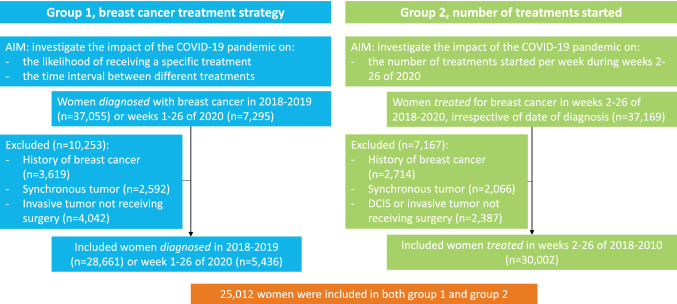
Flow chart of group 1 and 2

To answer aim one, women diagnosed with DCIS or invasive breast cancer between week 1 of 2018 and week 26 of 2020 were selected (group 1) (Fig. 2). Women diagnosed in 2020 were grouped into four, based on their date of diagnosis: pre-COVID (weeks 1–8), transition (weeks 9–12), lockdown (weeks 13–17), and care restart (weeks 18–26) (Fig. 1). Data of patients diagnosed in 2018/2019 (complete years) was included as a reference. Logistic regression was used to investigate the association between the period of diagnosis and likelihood of receiving: (1) surgery in patients with DCIS, (2) (neo-)adjuvant chemotherapy in patients with an invasive tumor, (3) (neo-)adjuvant endocrine therapy in patients with an HR+ invasive tumor, (4) (neo-)adjuvant targeted therapy in patients with an HER2+ invasive tumor, (5) mastectomy (compared to breast-conserving surgery) in patients with an invasive tumor, (6) IBR with autologous tissue, IBR with an implant, or IBR with autologous tissue and an implant in patients with an invasive tumor receiving mastectomy, and (7) chemotherapy after radiotherapy (compared to radiotherapy after chemotherapy) in patients with an invasive tumor receiving adjuvant chemotherapy and radiotherapy. Analyses were stratified by stage and tumor subtype, and adjusted for age (< 50, 50–74, > 74), stage, and tumor subtype. The analyses including patients with DCIS were further stratified by tumor grade (I–II or III). The group of patients diagnosed during lockdown or care restart barely included patients with a screen-detected tumor (due to the discontinuation of the screening program). A sensitivity analyses was performed excluding all patients with a screen-detected tumor to ensure that we were looking at changes in treatments instead of changes in patient groups.

To determine the time interval for patients with DCIS grade I–II, and DCIS grade III, median time [± 95% confidence interval (95%CI)] between diagnosis and surgery was calculated. For patients with an invasive tumor, median time (± 95%CI) between the following time points was calculated per tumor stage: (1) diagnosis and surgery (no neo-adjuvant treatment was given), (2) diagnosis and start of neo-adjuvant treatment, (3) diagnosis and start of first treatment (of any kind), (4) end of neo-adjuvant treatment and surgery, (5) surgery and start of adjuvant systemic therapy, and (6) surgery and start of adjuvant radiotherapy. Time intervals were calculated for each period of 2020 and compared with 2018/2019 using Wilcoxon rank-sum test.

#### Aim 2: impact on number of treatments started

To answer aim two, women treated for DCIS or invasive breast cancer in weeks 2–26 of 2018, 2019, or 2020 were selected (group 2). Women not receiving any treatment for DCIS were excluded. Each week started on a Monday, meaning that week one did not always include seven days, therefore, week one was omitted. To reduce random variation, the three-week moving average was calculated per treatment for each week of 2018, 2019, and 2020. The 3-week moving average shows the average number of patients starting a certain treatment per week during the week of interest and the 2 weeks preceding this week, starting with week 4. Subsequently, the 3-week moving average of 2020 was expressed as percentage of the corresponding three-week moving average of 2018/2019 (averaged).

A two-sided *p*-value < 0.05 was considered statistically significant. All data were analyzed using STATA version 17.0 (StataCorp, College Station, Texas, USA).

## Results

### Aim 1: breast cancer treatment strategy

A total of 3828 DCIS and 24,833 invasive breast tumors were diagnosed in 2018/2019, 344 DCIS and 2003 invasive tumors pre-COVID (weeks 1–8, 2020), 133 DCIS and 887 invasive tumors during transition (weeks 9–12), 51 DCIS and 535 invasive tumors during lockdown (weeks 13–17), and 111 DCIS and 1372 invasive tumors during care restart (weeks 18–26) (Table 2, group 1).

**Table 2:** Baseline characteristics of patients diagnosed or treated in 2018, 2019, and 2020 (N, %)

	Group 1 Patients diagnosed during:^a^					Group 2 Patients treated in weeks 2 to 26 of:^b^		
	2018/2019	Pre-COVID	Transition	Lockdown	Care restart	2018	2019	2020
Patients	28,661	2,347	1,020	586	1,483	10,785	10,875	9,596
Age group								
< 50	6,035 (21.1)	488 (20.8)	206 (20.2)	180 (30.7)	466 (31.4)	2,787 (25.8)	2,797 (25.7)	2,579 (26.9)
50–74	19,236 (67.1)	1,553 (66.2)	700 (68.6)	318 (54.3)	760 (51.3)	7,029 (65.2)	7,017 (64.5)	6,049 (63.0)
> 74	3,390 (11.8)	306 (13.0)	114 (11.2)	88 (15.0)	257 (17.3)	969 (9.0)	1,061 (9.8)	968 (10.1)
Stage								
DCIS, grade I–II	2,068 (7.2)	186 (7.9)	72 (7.1)	26 (4.4)	59 (4.0)	479 (4.4)	526 (4.8)	381 (4.0)
DCIS, grade III	1,561 (5.4)	133 (5.7)	55 (5.4)	22 (3.8)	43 (2.9)	463 (4.3)	449 (4.6)	348 (3.6)
DCIS, grade unknown	199 (0.7)	25 (1.1)	6 (0.6)	3 (0.5)	9 (0.6)	35 (0.3)	42 (0.4)	47 (0.5)
I	12,191 (42.5)	974 (41.5)	436 (42.8)	203 (34.6)	521 (35.1)	4,068 (37.7)	3,947 (36.3)	3,391 (35.3)
II	9,835 (34.3)	800 (34.1)	341 (33.4)	244 (41.6)	650 (43.8)	4,282 (39.7)	4,431 (40.7)	4,008 (41.8)
III	2,462 (8.6)	205 (8.7)	92 (9.0)	78 (13.3)	176 (11.9)	1,280 (11.9)	1,313 (12.1)	1,252 (13.1)
IV	334 (1.2)	24 (1.0)	17 (1.7)	10 (1.7)	25 (1.7)	173 (1.6)	163 (1.5)	167 (1.7)
Unknown	11 (0.0)	0 (0.0)	1 (0.1)	0 (0.0)	0 (0.0)	5 (0.1)	4 (0.0)	2 (0.0)
Screening status								
Screen-detected	11,914 (41.6)	949 (40.4)	467 (45.8)	67 (11.4)	32 (2.2)	4,065 (37.7)	4,006 (36.8)	2,987 (31.1)
Non-screen-detected	15,940 (55.6)	1,322 (56.3)	515 (50.5)	503 (85.8)	1,410 (95.1)	6,425 (59.6)	6,533 (60.1)	6,255 (65.2)
Unknown	807 (2.8)	76 (3.2)	38 (3.7)	16 (2.2)	41 (2.8)	295 (2.7)	336 (3.1)	354 (3.7)
Hormone receptor status^c^								
Positive	20,739 (83.5)	1,666 (83.2)	742 (83.7)	424 (79.3)	1,107 (80.7)	8,019 (81.8)	8,017 (81.3)	7,059 (80.0)
Negative	3,949 (15.9)	320 (16.0)	136 (15.3)	107 (20.0)	254 (18.5)	1,750 (17.8)	1,799 (18.3)	1,708 (19.4)
Unknown	145 (0.6)	17 (0.9)	9 (1.0)	4 (0.8)	11 (0.8)	39 (0.4)	42 (0.4)	53 (0.6)
HER2 status^c^								
Positive	3,330 (13.4)	227 (11.3)	104 (11.7)	89 (16.6)	212 (15.5)	1,614 (16.5)	1,561 (15.8)	1,430 (16.2)
Negative	20,994 (84.5)	1,734 (86.6)	765 (86.3)	437 (81.7)	1,123 (81.9)	8,055 (82.1)	8,149 (82.7)	7,246 (82.2)
Unknown	509 (2.1)	42 (2.1)	18 (2.0)	9 (1.7)	37 (2.7)	139 (1.4)	148 (1.5)	144 (1.6)
Subtype^c^								
HR+/HER2+	2,277 (9.2)	160 (8.0)	70 (7.9)	59 (11.0)	145 (10.6)	1,120 (11.4)	1,090 (11.1)	995 (11.3)
HR+/HER2−	18,136 (73.0)	1,484 (74.1)	665 (75.0)	362 (67.8)	938 (68.4)	6,804 (69.4)	6,833 (69.3)	5,982 (67.8)
HR−/HER2+	1,049 (4.2)	67 (3.3)	34 (3.8)	30 (5.6)	67 (4.9)	494 (5.0)	471 (4.8)	434 (4.9)
HR−/HER2−	2,854 (11.5)	249 (12.4)	100 (11.3)	75 (14.0)	185 (13.5)	1,249 (12.7)	1,313 (13.3)	1,263 (14.3)
Unknown	517 (2.1)	43 (2.2)	18 (2.0)	9 (1.7)	37 (2.7)	141 (1.4)	151 (1.5)	146 (1.7)
Patients with DCIS grade I or II receiving surgery	1,758 (85.0)	148 (79.6)	49 (68.1)	23 (88.5)	42 (71.2)	NA	NA	NA
Patients with DCIS grade III receiving surgery	1,533 (98.2)	129 (97.0)	55 (100.0)	21 (95.5)	43 (100.0)	NA	NA	NA
Neo-adjuvant chemotherapy^d^	6,142 (24.7)	492 (24.6)	167 (18.8)	187 (35.0)	483 (35.2)	1,381 (12.8)	1,534 (14.1)	1,335 (13.9)
Neo-adjuvant endocrine therapy^d^	1,120 (4.5)	137 (6.8)	105 (11.8)	47 (8.8)	92 (6.7)	233 (2.2)	286 (2.6)	411 (4.3)
Neo-adjuvant targeted therapy^d^	2,001 (8.1)	150 (7.5)	58 (6.5)	68 (12.7)	160 (11.7)	425 (3.9)	471 (4.3)	442 (4.6)
Breast-conserving surgery^d^	17,136 (69.0)	1,398 (69.8)	605 (68.2)	341 (63.7)	822 (59.9)	4,766 (44.2)	4,932 (45.4)	4,100 (42.7)
Mx^d^	7,697 (31.0)	605 (30.2)	282 (31.8)	194 (36.3)	550 (40.1)	2,160 (20.0)	2,104(19.4)	1,960 (20.4)
Mx and IBR^d^	2,321 (9.4)	176 (8.8)	70 (7.9)	49 (9.2)	207 (15.1)	710 (6.6)	677 (6.2)	641 (671)
Mx and IBR with autologous tissue^d^	232 (0.9)	19 (1.0)	5 (0.6)	4 (0.8)	36 (2.6)	118 (1.1)	80 (0.7)	73 (0.8)
Mx and IBR with implant^d^	1,950 (7.9)	144 (7.2)	60 (6.8)	38 (7.1)	156 (11.4)	554 (5.1)	574 (5.3)	516 (5.4)
Mx and IBR with autologous tissue and implant^d^	90 (0.4)	2 (0.1)	1 (0.1)	3 (0.6)	5 (0.4)	33 (0.3)	16 (0.2)	20 (0.2)
Adjuvant chemotherapy^d^	4,215 (17.0)	336 (16.8)	189 (21.3)	101 (18.9)	267 (19.5)	1,054 (9.8)	935 (8.6)	1,017 (10.6)
Adjuvant endocrine therapy^d^	12,805 (51.6)	1,019 (50.9)	470 (53.0)	295 (55.1)	791 (57.6)	2,945 (27.3)	2,911 (26.8)	2,815 (29.3)
Adjuvant targeted therapy^d^	2,802 (11.3)	204 (10.2)	92 (10.4)	77 (14.4)	183 (13.3)	350 (3.3)	369 (3.4)	553 (5.8)
Adjuvant radiotherapy^d^	18,971 (76.4)	1,506 (75.2)	671 (75.7)	410 (76.6)	989 (72.1)	4,989 (46.3)	4,943 (45.5)	4,731 (49.3)
Adjuvant chemotherapy-radiotherapy sequence^d^								
Chemotherapy first	1,063 (30.7)	50 (17.7)	35 (22.7)	19 (22.4)	69 (30.1)	NA	NA	NA
Radiotherapy first	2,351 (67.8)	229 (80.9)	116 (75.3)	60 (70.6)	156 (68.1)	NA	NA	NA
Concurrent	40 (1.2)	3 (1.1)	2 (1.3)	5 (5.9)	1 (0.0)	NA	NA	NA
Unknown	14 (0.4)	1 (0.4)	1 (0.7)	1 (1.2)	3 (1.3)	NA	NA	NA

Pre-COVID: weeks 1–8, 2020; Transition: weeks 9–12, 2020; Lockdown: weeks 13–17, 2020; Care restart: weeks 18–26, 2020

*HER2* Human epidermal growth receptor 2, *HR* Hormone receptor, *IBR* Immediate breast reconstruction, *Mx* Mastectomy, *NA* Not applicable

^a^Patients diagnosed with DCIS or invasive tumor in 2018/2019 or weeks 1 to 26 of 2020, excluding patients not receiving surgery for their invasive tumor

^b^Patients treated for their DCIS or invasive tumor in weeks 2–26 of 2018, 2019, or 2020, excluding patients not receiving surgery for their DCIS or invasive tumor. Patients can receive multiple treatments in one year and patients can receive treatment in both 2018, 2019 and 2020, and can therefore be part of multiple treatment years. For each treatment the number of patients receiving that treatment in weeks 2–26 of 2018, 2019, or 2020 are shown

^c^For this variable only patients with an invasive tumor were included

^d^For this variable only patients with an invasive tumor were included in group 1

### Likelihood of treatment

#### Neo-adjuvant treatment

Compared to 2018/2019, neo-adjuvant chemotherapy was less likely for patients diagnosed during transition with a stage I, stage II, HR+/HER2−, or HR−/HER2− tumor (odds ratio (OR) 0.24, 95%CI 0.11–0.53; OR 0.63, 95%CI 0.47–0.68; OR 0.55, 95%CI 0.41–0.75; OR 0.46, 95%CI 0.27–0.77, respectively), while neo-adjuvant chemotherapy was more likely for patients diagnosed during care restart with a stage I, stage II, HR+/HER2+, or HR−/HER2− (OR 1.88, 95%CI 1.29–2.75; OR 1.36, 95%CI 1.11–1.68; OR 2.11, 95%CI 1.32–3.38; OR 2.49, 95%CI 1.55–4.00, respectively) (Tables 3 and 4). The sensitivity analysis, on 19,690 patients with a non-screen-detected tumor, showed that these associations were no longer present in patients diagnosed during transition or care restart with a stage II tumor (Supplementary Tables 1 and 2).

**Table 3:** Logistic regression calculating the odds ratios and 95% confidence interval of the association between period of diagnosis and likelihood of receiving a specific treatment, stratified by stage (group 1)

	Pre-COVID	Transition	Lockdown	Care restart
DCIS, grade I–II				
Surgery	0.69 (0.47–1.02)	0.36 (0.21–0.60)	1.39 (0.41–4.74)	0.45 (0.25–0.82)
DCIS, grade III				
Surgery	0.70 (0.23–2.09)	NA	0.38 (0.05–3.12)	NA
Stage I				
Neo-adjuvant chemotherapy	1.36 (0.97–1.91)	0.24 (0.11–0.53)	2.08 (1.15–3.73)	1.88 (1.29–2.75)
Neo-adjuvant endocrine therapy^a^	2.43 (1.55–3.82)	5.10 (3.13–8.29)	5.05 (2.59–9.86)	1.45 (0.70–3.00)
Neo-adjuvant targeted therapy^b^	1.51 (0.86–2.66)	0.32 (0.10–1.07)	2.52 (0.94–6.75)	1.50 (0.83–2.70)
Mastectomy	0.88 (0.73–1.06)	1.06 (0.82–1.37)	1.12 (0.79–1.59)	1.23 (0.99–1.53)
Adjuvant chemotherapy	0.91 (0.72–1.17)	1.35 (0.98–1.86)	0.85 (0.52–1.40)	0.91 (0.67–1.25)
Adjuvant endocrine therapy^a^	0.99 (0.85–1.15)	1.06 (0.86–1.32)	1.37 (1.00–1.87)	1.24 (1.02–1.52)
Adjuvant targeted therapy^b^	1.96 (0.96–4.03)	0.70 (0.33–1.51)	1.11 (0.33–3.69)	0.77 (0.39–1.50)
Adjuvant radiotherapy	0.93 (0.79–1.08)	0.88 (0.70–1.09)	1.11 (0.79–1.54)	0.85 (0.70–1.04)
IBR with autologous tissue^c^	0.83 (0.33–2.08)	NA	NA	2.90 (1.55–5.45)
IBR with implant^c^	0.94 (0.65–1.36)	1.13 (0.68–1.88)	0.89 (0.45–1.75)	1.18 (0.77–1.79)
IBR with autologous tissue and implant^c^	0.44 (0.06–3.23)	NA	NA	0.51 (0.07–3.78)
Chemotherapy after radiotherapy^d^	1.48 (0.78–2.81)	3.56 (1.08–11.71)	0.82 (0.26–2.64)	0.74 (0.40–1.39)
Stage II				
Neo-adjuvant chemotherapy	1.00 (0.83–1.22)	0.63 (0.47–0.86)	1.08 (0.77–1.51)	1.36 (1.11–1.68)
Neo-adjuvant endocrine therapy^a^	1.59 (1.25–2.03)	3.09 (2.30–4.14)	1.34 (0.85–2.11)	1.54 (1.18–2.02)
Neo-adjuvant targeted therapy^b^	1.25 (0.76–2.05)	1.01 (0.49–2.07)	1.61 (0.70–3.69)	2.77 (1.51–5.12)
Mastectomy	1.00 (0.86–1.17)	0.96 (0.76–1.21)	0.95 (0.73–1.25)	1.21 (1.03–1.43)
Adjuvant chemotherapy	1.04 (0.87–1.25)	1.53 (1.19–1.96)	1.18 (0.86–1.61)	0.96 (0.78–1.17)
Adjuvant endocrine therapy^a^	1.00 (0.78–1.28)	1.10 (0.75–1.62)	0.74 (0.50–1.10)	1.00 (0.76–1.31)
Adjuvant targeted therapy^b^	1.30 (0.63–2.67)	7.31 (0.85–63.10)	1.54 (0.50–4.74)	1.71 (0.81–3.61)
Adjuvant radiotherapy	0.94 (0.79–1.12)	1.06 (0.81–1.38)	1.06 (0.78–1.44)	0.86 (0.72–1.03)
IBR with autologous tissue^c^	1.22 (0.63–2.36)	1.15 (0.42–3.21)	0.80 (0.19–3.32)	1.96 (1.13–3.38)
IBR with implant^c^	0.89 (0.65–1.20)	0.64 (0.38–1.06)	0.38 (0.20–0.75)	1.15 (0.85–1.55)
IBR with autologous tissue and implant^c^	NA	0.69 (0.09–5.08)	2.86 (0.86–9.51)	0.79 (0.24–2.57)
Chemotherapy after radiotherapy^d^	2.15 (1.37–3.36)	1.32 (0.78–2.23)	1.23 (0.63–2.41)	1.04 (0.68–1.59)
Stage III				
Neo-adjuvant chemotherapy	0.79 (0.55–1.14)	0.63 (0.38–1.04)	1.73 (0.86–3.47)	1.33 (0.86–2.04)
Neo-adjuvant endocrine therapy^a^	1.12 (0.68–1.83)	1.78 (0.96–3.30)	2.44 (1.26–4.72)	0.77 (0.41–1.43)
Neo-adjuvant targeted therapy^b^	0.50 (0.21–1.19)	1.03 (0.28–3.72)	8.46 (0.99–72.40)	1.04 (0.31–3.44)
Mastectomy	0.81 (0.60–1.10)	1.59 (0.96–2.65)	0.95 (0.58–1.55)	1.24 (0.87–1.77)
Adjuvant chemotherapy	1.19 (0.85–1.66)	1.50 (0.93–2.43)	0.79 (0.44–1.42)	1.37 (0.96–1.94)
Adjuvant endocrine therapy^a^	0.59 (0.35–0.99)	0.92 (0.39–2.20)	0.48 (0.23–1.03)	2.00 (0.80–5.01)
Adjuvant targeted therapy^b^	1.21 (0.35–4.16)	2.74 (0.52–14.39)	2.07 (0.40–10.77)	0.80 (0.20–3.19)
Adjuvant radiotherapy	1.20 (0.70–2.06)	0.76 (0.39–1.49)	0.85 (0.39–1.83)	0.90 (0.52–1.58)
IBR with autologous tissue^c^	1.60 (0.47–5.41)	NA	2.46 (0.56–10.84)	2.40 (0.89–6.45)
IBR with implant^c^	1.03 (0.58–1.83)	0.76 (0.32–1.82)	1.25 (0.56–2.77)	1.22 (0.72–2.06)
IBR with autologous tissue and implant^c^	1.95 (0.24–16.15)	NA	NA	2.19 (0.26–18.30)
Chemotherapy after radiotherapy^d^	3.56 (1.80–7.04)	1.26 (0.55–2.90)	3.75 (0.96–14.64)	1.46 (0.77–2.78)
Stage IV				
Neo-adjuvant chemotherapy	0.81 (0.25–2.60)	0.70 (0.21–2.35)	2.08 (0.24–17.76)	1.38 (0.33–5.84)
Neo-adjuvant endocrine therapy^a^	1.26 (0.33–4.78)	2.18 (0.69–6.89)	NA	0.86 (0.18–4.16)
Neo-adjuvant targeted therapy^b^	NA	NA	NA	NA
Mastectomy	0.82 (0.35–1.90)	1.19 (0.43–3.34)	1.43 (0.36–5.68)	1.18 (0.50–2.78)
Adjuvant chemotherapy	0.59 (0.16–2.19)	0.36 (0.04–2.82)	0.84 (0.16–4.34)	2.72 (1.06–6.98)
Adjuvant endocrine therapy^a^	0.65 (0.13–3.14)	1.32 (0.16–10.64)	NA	1.10 (0.13–9.25)
Adjuvant targeted therapy^b^	NA	NA	NA	0.41 (0.04–4.41)
Adjuvant radiotherapy	0.99 (0.37–2.65)	1.23 (0.37–4.10)	1.06 (0.22–5.24)	1.44 (0.46–4.48)
IBR with autologous tissue^c^	NA	NA	NA	NA
IBR with implant^c^	NA	2.57 (0.46–14.51)	NA	1.61 (0.40–6.47)
IBR with autologous tissue and implant^c^	NA	NA	NA	NA
Chemotherapy after radiotherapy^d^	NA	NA	NA	3.84 (0.40–37.22)

*Reference* 2018/2019; Pre-COVID: weeks 1–8, 2020; Transition: weeks 9–12, 2020; Lockdown: weeks 13–17, 2020; Care restart: weeks 18–26, 2020**.** Adjusted for age (< 50, 50–74, > 74) and tumor subtype (HR+/HER2+, HR+/HER2−, HR−/HER2+, HR−/HER2−)

*HER2* Human epidermal growth receptor 2, *HR* Hormone receptor, *IBR* Immediate breast reconstruction, *NA* Too few patients for the analysis

^a^These analyses only included patients with an HR+tumor

^b^These analyses only included patients with an HER2+tumor

^c^These analyses only included patients treated with a mastectomy

^d^These analyses only included patients receiving adjuvant chemotherapy and radiotherapy. The likelihood of receiving chemotherapy after radiotherapy was compared with having radiotherapy after chemotherapy

**Table 4:** Logistic regression calculating the odds ratios and 95% confidence interval of the association between period of diagnosis and likelihood of receiving a specific treatment, stratified by tumor subtype (group 1)

	Pre-COVID	Transition	Lockdown	Care restart
HR+/HER2+				
Neo-adjuvant chemotherapy	1.10 (0.73–1.66)	0.72 (0.39–1.32)	2.85 (1.33–6.08)	2.11 (1.32–3.38)
Neo-adjuvant endocrine therapy^a^	1.03 (0.47–2.28)	2.56 (1.11–5.89)	3.54 (1.64–7.60)	1.13 (0.51–2.52)
Neo-adjuvant targeted therapy^b^	0.93 (0.62–1.38)	0.74 (0.41–1.34)	2.77 (1.33–5.78)	2.03 (1.29–3.20)
Mastectomy	0.85 (0.60–1.22)	1.37 (0.82–2.28)	0.77 (0.43–1.37)	1.09 (0.76–1.57)
Adjuvant chemotherapy	1.27 (0.88–1.83)	1.60 (0.94–2.73)	0.58 (0.27–1.21)	0.71 (0.46–1.10)
Adjuvant endocrine therapy^a^	0.81 (0.54–1.19)	1.81 (0.88–3.73)	1.04 (0.53–2.06)	0.92 (0.60–1.42)
Adjuvant targeted therapy^b^	1.34 (0.80–2.24)	1.84 (0.85–4.01)	1.53 (0.65–3.62)	1.20 (0.68–2.10)
Adjuvant radiotherapy	1.15 (0.76–1.73)	0.73 (0.42–1.26)	1.22 (0.61–2.41)	0.86 (0.58–1.27)
IBR with autologous tissue^c^	0.49 (0.07–3.73)	1.79 (0.40–8.01)	NA	1.35 (0.39–4.62)
IBR with implant^c^	1.50 (0.76–2.97)	0.83 (0.33–2.13)	1.62 (0.53–4.94)	1.27 (0.66–2.43)
IBR with autologous tissue and implant^c^	1.90 (0.23–15.78)	NA	8.02 (0.86–74.37)	NA
Chemotherapy after radiotherapy^d^	1.00 (0.49–2.04)	1.63 (0.49–5.44)	4.30 (0.39–47.00)	0.82 (0.33–2.00)
HR+/HER2−				
Neo-adjuvant chemotherapy	0.83 (0.68–1.02)	0.55 (0.41–0.75)	1.21 (0.86–1.70)	1.19 (0.96–1.46)
Neo-adjuvant endocrine therapy^a^	1.65 (1.35–2.02)	3.10 (2.44–3.94)	1.65 (1.15–2.37)	1.38 (1.08–1.76)
Neo-adjuvant targeted therapy^b^	NA	NA	NA	NA
Mastectomy	0.94 (0.82–1.07)	1.06 (0.88–1.27)	1.17 (0.93–1.49)	1.33 (1.14–1.54)
Adjuvant chemotherapy	1.00 (0.84–1.18)	1.20 (0.95–1.52)	1.08 (0.79–1.47)	1.09 (0.90–1.33)
Adjuvant endocrine therapy^a^	0.99 (0.87–1.12)	1.02 (0.84–1.24)	1.05 (0.80–1.37)	1.25 (1.05–1.48)
Adjuvant targeted therapy^b^	NA	NA	NA	NA
Adjuvant radiotherapy	0.95 (0.83–1.07)	0.96 (0.80–1.16)	0.92 (0.72–1.17)	0.81 (0.69–0.94)
IBR with autologous tissue^c^	1.20 (0.69–2.10)	0.35 (0.08–1.41)	0.78 (0.24–2.48)	2.38 (1.53–3.70)
IBR with implant^c^	0.87 (0.67–1.13)	0.97 (0.68–1.39)	0.62 (0.38–0.99)	1.19 (0.92–1.53)
IBR with autologous tissue and implant^c^	0.20 (0.03–1.46)	0.44 (0.06–3.17)	0.67 (0.09–4.87)	1.12 (0.44–2.81)
Chemotherapy after radiotherapy^d^	2.60 (1.71–3.97)	1.17 (0.72–1.91)	1.21 (0.63–2.34)	1.14 (0.77–1.67)
HR−/HER2+				
Neo-adjuvant chemotherapy	2.87 (1.28–6.44)	0.78 (0.31–1.97)	1.63 (0.55–4.82)	1.66 (0.80–3.44)
Neo-adjuvant endocrine therapy^a^	NA	NA	NA	NA
Neo-adjuvant targeted therapy^b^	2.99 (1.34–6.68)	0.82 (0.33–2.05)	1.70 (0.58–5.01)	1.75 (0.85–3.60)
Mastectomy	0.79 (0.47–1.35)	0.80 (0.38–1.67)	0.81 (0.37–1.76)	1.07 (0.64–1.79)
Adjuvant chemotherapy	0.58 (0.28–1.21)	1.11 (0.47–2.64)	0.44 (0.14–1.42)	0.62 (0.29–1.29)
Adjuvant endocrine therapy^a^	NA	NA	NA	NA
Adjuvant targeted therapy^b^	3.18 (0.98–10.30)	0.77 (0.26–2.22)	1.22 (0.31–4.80)	0.74 (0.33–1.67)
Adjuvant radiotherapy	1.51 (0.79–2.90)	1.50 (0.60–3.75)	0.95 (0.39–2.33)	1.15 (0.63–2.10)
IBR with autologous tissue^c^	2.62 (0.51–13.45)	NA	2.38 (0.25–22.79)	0.85 (0.10–7.14)
IBR with implant^c^	0.40 (0.11–1.44)	0.81 (0.16–4.16)	0.45 (0.09–2.30)	0.63 (0.22–1.77)
IBR with autologous tissue and implant^c^	NA	NA	NA	NA
Chemotherapy after radiotherapy^d^	4.41 (0.43–45.48)	2.82 (0.52–15.33)	NA	0.27 (0.04–1.84)
HR−/HER2−				
Neo-adjuvant chemotherapy	1.54 (1.07–2.21)	0.46 (0.27–0.77)	0.95 (0.50–1.81)	2.49 (1.55–4.00)
Neo-adjuvant endocrine therapy^a^	NA	NA	NA	NA
Neo-adjuvant targeted therapy^b^	NA	NA	NA	NA
Mastectomy	1.02 (0.77–1.37)	0.98 (0.62–1.54)	0.78 (0.46–1.31)	0.97 (0.69–1.35)
Adjuvant chemotherapy	1.07 (0.80–1.42)	2.87 (1.85–4.45)	1.30 (0.80–2.11)	1.25 (0.91–1.72)
Adjuvant endocrine therapy^a^	NA	NA	NA	NA
Adjuvant targeted therapy^b^	NA	NA	NA	NA
Adjuvant radiotherapy	0.74 (0.54–1.01)	0.84 (0.52–1.37)	2.64 (1.19–5.88)	1.16 (0.78–1.73)
IBR with autologous tissue^c^	0.49 (0.06–3.69)	NA	NA	3.16 (1.15–8.68)
IBR with implant^c^	1.00 (0.55–1.80)	0.28 (0.08–1.03)	0.49 (0.13–1.77)	1.35 (0.73–2.50)
IBR with autologous tissue and implant^c^	NA	NA	5.22 (0.59–46.13)	NA
Chemotherapy after radiotherapy^d^	3.05 (1.20–7.71)	3.18 (0.97–10.40)	1.24 (0.41–3.73)	1.27 (0.63–2.56)

*Reference* 2018/2019; Pre-COVID: weeks 1–8, 2020; Transition: weeks 9–12, 2020; Lockdown: weeks 13–17, 2020; Care restart: weeks 18–26, 2020. Adjusted for age (< 50, 50–74, > 74) and stage

*HER2* Human epidermal growth receptor 2, *HR* Hormone receptor, *IBR* Immediate breast reconstruction, *NA* Not applicable or too few patients for the analysis

^a^These analyses only included patients with an HR+tumor

^b^These analyses only included patients with an HER2+tumor

^c^These analyses only included patients treated with a mastectomy

^d^These analyses only included patients receiving adjuvant chemotherapy and radiotherapy. The likelihood of receiving chemotherapy after radiotherapy was compared with having radiotherapy after chemotherapy

Neo-adjuvant endocrine therapy was more likely for patients diagnosed during transition with a stage I, stage II, HR+/HER2+, or HR+/HER2− tumor (OR 5.10, 95%CI 3.13–8.29; OR 3.09, 95%CI 2.30–4.14; OR 2.56, 95%CI 1.11–5.89; OR 3.10, 95%CI 2.44–3.94, respectively), or during lockdown with a stage I, stage III, HR+/HER2+, or HR+/HER2− tumor (OR 5.05, 95%CI 2.95–9.86; OR 2.44, 95%CI 1.26–4.72; OR 3.54, 95%CI 1.64–7.60; OR 1.65, 95%CI 1.15–2.37, respectively). In the sensitivity analysis, this association was no longer present in patients diagnosed during transition with a HR+/HER2+tumor.

#### Surgery

Surgery was less likely for patients diagnosed with DCIS, grade I–II, during transition or care restart (OR 0.36, 95%CI 0.21–0.60; OR 0.45, 95%CI 0.25–0.82, respectively). These associations were still present in the sensitivity analysis. A mastectomy was more likely for patients diagnosed during care restart with a stage II or HR+/HER2− tumor (OR 1.21, 95%CI 1.03–1.43; OR 1.33, 95%CI 1.14–1.54, respectively). These associations were no longer present in the sensitivity analysis. An IBR with autologous tissue was more likely for patients treated with a mastectomy and diagnosed during transition with a stage I, stage II, HR+/HER2−, or HR−/HER2− tumor (OR 2.90, 95%CI 1.55–5.45; OR 1.96, 95%CI 1.13–3.38; OR 2.38, 95%CI 1.53–3.70; OR 3.16, 95%CI 1.15–8.68, respectively). These associations were still present in the sensitivity analysis.

#### Adjuvant treatment

Adjuvant chemotherapy was more likely for patients diagnosed during transition with a stage II or HR−/HER2− tumor (OR 1.53, 95%CI 1.19–1.96, OR 2.87, 95%CI 1.85–4.45, respectively). Chemotherapy after radiotherapy was more likely for patients diagnosed pre-COVID with a stage II, stage III, HR+/HER2−, or HR−/HER2− tumor (OR 2.15, 95%CI 1.37–3.36; OR 3.56, 95%CI 1.80–7.04; OR 2.60, 95%CI 1.71–3.97; OR 3.05, 95%CI 1.20–7.71, respectively). All these associations were still present in the sensitivity analysis.

#### Time intervals

**Fig. 3:**
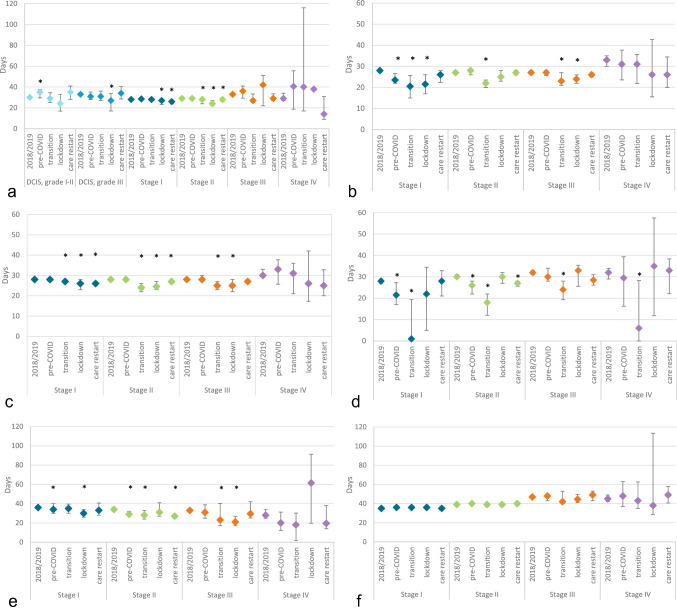
Time interval (median, 95% confidence interval) between **a** diagnosis and surgery (excluding patients with an invasive tumor receiving neo-adjuvant treatment), **b** diagnosis and start of neo-adjuvant treatment, **c** diagnosis and start of first treatment (of any kind), **d** end of neo-adjuvant treatment and surgery, **e** surgery and start of adjuvant treatment, and **f** surgery and start of radiotherapy, for patients diagnosed between week 1 of 2018 and week 26 of 2020 (group 1). Time intervals are stratified by period of diagnosis and tumor stage. Pre-COVID: weeks 1–8, 2020; Transition: weeks 9–12, 2020; Lockdown: weeks 13–17, 2020; Care restart: weeks 18–26, 2020. *significantly different time interval in that period of 2020 compared to 2018/2019 (*p* < 0.05)

Compared to 2018/2019, the median time interval between diagnosis and surgery increased from 30 days (95%CI 29–31) to 35 days (95%CI 30–37) for patients diagnosed pre-COVID with a DCIS, grade I–II (*p* = 0.035), and decreased from 33 days (95%CI 32–34) to 27 days (95%CI 17–34) for patients diagnosed during lockdown with a DCIS, grade III (*p* = 0.024) (Fig. 3). Median time interval between diagnosis and first treatment decreased for patients diagnosed during lockdown with a stage I, II, or III tumor [from 28 (95%CI 28–28] to 26 days (95%CI 25–27), *p* = 0.002; from 28 (95%CI 28–28) to 24.5 days (95%CI 23–27), *p* < 0.001; from 28 (95%CI 28–28) to 25 days (95%CI22–28), *p* = 0.006, respectively).

### Aim 2: number of treatments started

**Fig. 4:**
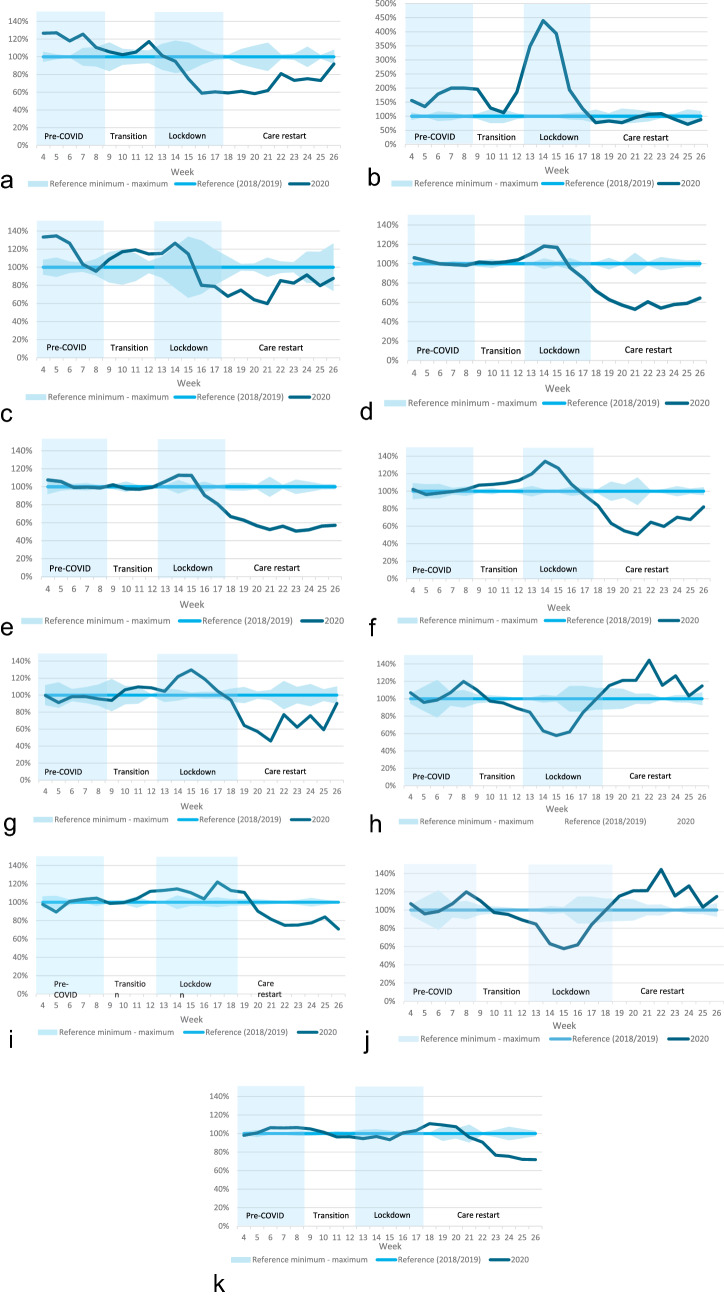
Percentage change in the 3-week moving average of the number of patients (group 2) starting **a** neo-adjuvant chemotherapy, **b** neo-adjuvant endocrine therapy, **c** neo-adjuvant targeted therapy, **d** surgery, **e** breast-conserving surgery, **f** mastectomy, **g** mastectomy with immediate breast reconstruction, **h** adjuvant chemotherapy, **i** adjuvant endocrine therapy, **j** adjuvant targeted therapy, or **k** adjuvant radiotherapy. 3-week moving average was calculated over the week of interest and the two weeks preceding this week Percentage change calculated as the percentage of the corresponding three-week moving average of 2018/2019

A total of 21,660 women were treated in weeks 2–26 of 2018/2019, and 9,596 in weeks 2–26 of 2020 (Table 2, group 2). Compared to the corresponding three-week moving average of 2018/2019, the number of patients who started neo-adjuvant endocrine therapy in week 14 of 2020 increased by 339% (Fig. 4). The number of surgeries, breast-conserving surgeries, and mastectomies performed in week 14 increased by 18%, 13%, and 34%, respectively. The number of adjuvant chemotherapies started per week decreased by 42% in week 15 and increased by 44% in week 22. The use of radiotherapy started to decrease from week 21 onwards, with 23% fewer patients starting radiotherapy in week 23.

## Discussion

The COVID-19 pandemic and the subsequently altered recommendations had a significant impact on various parts of the breast cancer treatment strategy and on the number of treatments started per week in the Netherlands. Patients diagnosed at the start of the pandemic were more likely to receive neo-adjuvant endocrine therapy, while they were less likely to receive neo-adjuvant chemotherapy. In general time intervals between treatments decreased. At the start of the pandemic, the number of neo-adjuvant endocrine therapies and surgeries started per week increased, while the number of adjuvant chemotherapies therapies started per week decreased. These adaptations showed that the recommendations were partly implemented in daily practice.

### Aim 1: breast cancer treatment strategy

According to the recommendation [4], patients diagnosed during transition with a stage I, stage II, HR+/HER2−, or HR−/HER2− tumor, were less likely to receive neo-adjuvant chemotherapy, probably because it was thought that chemotherapy would increase the risk of COVID-19 related complications. In HR+/HER2− patients neo-adjuvant chemotherapy could be replaced by neo-adjuvant endocrine therapy. In HR−/HER2− patients neo-adjuvant chemotherapy was probably replaced by surgery as first treatment. Surgery was probably favored because of rising concerns that it might not be possible to perform surgery in the near future due to an increasing number of hospitalized COVID-19 patients. Patients diagnosed during transition with a stage II or HR−/HER2− tumor were more likely to receive adjuvant chemotherapy, suggesting that the timing of chemotherapy had been changed from before to after surgery. Patients diagnosed during care restart had an increased likelihood of receiving neo-adjuvant chemotherapy, which could partly be explained by study results published in week 22 showing no association between chemotherapy and mortality in COVID-19 patients [17], and because of less concerns about limited surgery facilities. Previous meta-analyses found no difference in survival between patients receiving neo-adjuvant or adjuvant chemotherapy [18, 19]. Moreover, the current study, just as studies performed in the United Kingdom and United States [12, 14, 20], showed that the recommendation to use neo-adjuvant endocrine therapy to postpone surgery was quickly implemented [4–7]. Previous research concluded that neo-adjuvant endocrine treatment can safely be used in some patients (e.g., postmenopausal, early stage, estrogen receptor-positive, and HER2−negative) to delay surgery for at least 6 months [21].

According to the recommendations [5–7], our results showed an increase, in the time interval between diagnosis and surgery for patients diagnosed pre-COVID with DCIS, grade I–II. We also showed that patients diagnosed with a low-grade DCIS were less likely to undergo surgery. As recently more attention is being paid to the de-escalation of treatment for low-grade DCIS, these findings can probably not solely be attributed to the effect of the COVID-19 pandemic [22, 23]. The recommendation to avoid IBR with autologous tissue [6, 8] or IBR altogether [4] was implemented in Italy and the United Kingdom [12, 24]. Our study showed that patients diagnosed during care restart had an increased likelihood of receiving IBR with autologous tissue. Closer examination showed that the percentage of patients with an IBR with autologous tissue receiving neo-adjuvant therapy did not differ between patients diagnosed during care restart and patients diagnosed in 2018/2019. Therefore, it was thought that the increased likelihood of receiving IBR with autologous tissue could be due to the reduction in the number of patients, the postponement of elective surgeries, and the increased availability of plastic surgeons due to cancellation of elective non-oncological surgeries. Consistent with the recommendations [6], specific patients diagnosed pre-COVID were more likely to receive chemotherapy after radiotherapy. This order was probably chosen as it allowed postponement of chemotherapy.

The sensitivity analysis, only including patients with a non-screen-detected tumor, showed that patients with a non-screen-detected tumor diagnosed during care restart were no longer more likely to receive a mastectomy. This might suggest that patients with a non-screen-detected tumor were more likely to receive a mastectomy compared to patients with a screen-detected tumor, probably due to different tumor characteristics.

The prioritization of oncological care, the reduction in the number of patients, and the postponement of elective surgeries probably led to a shorter time interval between various treatments for patients diagnosed with a DCIS, grade III, or stage I, II, or III tumor. A Canadian study also showed a reduction in the time interval between diagnosis and surgery [25]. A study of the United States showed no increase in the interval between diagnosis and first treatment [20]. An Italian study showed an increase in the interval between diagnosis and surgery, most likely because of the reorganization of the health care system, the redistribution of resources [13], and the high COVID-19 incidence in Italy at the pandemic outbreak.

### Aim 2: number of treatments started

The peak in the number of surgeries at the start of the pandemic is probably the result of the prioritization of oncological care and the postponement of elective surgeries. The reasons for the decrease and increase in the use of adjuvant chemotherapy are described above for neo-adjuvant chemotherapy. The decrease in the use of radiotherapy from week 21 onwards can be explained by the decrease in the number of breast cancer surgeries from week 16 onwards, in combination with the median time interval between surgery and radiotherapy of five weeks. An English study also showed a decrease in the number of patients starting radiotherapy two to three months after the start of the lockdown [26].

A Scottish and English study reported a decrease in the use of systemic anticancer treatments at the start of the pandemic [27, 28]. Our results did not indicate a large decline in the number of systemic anticancer treatments, as the decrease in the use of chemotherapy was compensated by the increase in the use of endocrine therapy. However, it is hard to compare the results of these studies with our results, as they did not separate the different systemic treatments.

### Strong points and limitations

This study benefited from the use of data from the NCR, including data on all women diagnosed with breast cancer in the Netherlands, thereby accurately reflecting daily practice. This study also has its limitations. First, it was only known when a patient started a specific treatment, making it impossible to investigate the total number of treatments per week. Second, 632 patients (2.1% of the patients with an invasive tumor) were excluded from the logistic regression analyses because of missing data on tumor stage and/or subtype. Since this is a very low percentage it is not expected that this has affected the results. Third, compared to 2018/2019, a higher number of patients started neo-adjuvant chemotherapy, neo-adjuvant endocrine therapy, and (neo-)adjuvant targeted therapy in the weeks preceding the pandemic. This shows that fluctuations and trends in treatment strategy unrelated to the COVID-19 pandemic and the subsequently altered treatment recommendations cannot be excluded. Therefore, results should be interpreted with care. Fourth, some of the patient groups include a low number of patients, such as the group of patients diagnosed during lockdown with a stage IV tumor. This might have resulted in a limited power for the analyses involving these patients.

## Conclusion

Our study showed that the breast cancer treatment strategy and the number of treatments started per week were quickly adapted during the COVID-19 pandemic, reflecting the resilience of the Dutch breast cancer care. Part of the COVID-19 related treatment recommendations were quickly implemented in clinical practice in the Netherlands, especially the recommendation to postpone surgery in case of DCIS, grade I–II, and to start neo-adjuvant endocrine therapy. This quick implementation was probably due to short communication lines between the care givers through national scientific associations. The current study suggests that the order of treatments has mainly been adapted, allowing breast cancer patients to still receive all essential treatments. A decrease in time intervals was shown, probably due to the successful prioritization of oncological care, a decrease in the number of patients, and the postponement of elective surgeries. We believe that the Dutch hospitals responded adequality to the COVID-19 pandemic, adapting treatment strategies in a way that decreased the risk of patients getting COVID-19 (related complications), without delaying treatment. Future studies investigating the consequences of those changes in the treatment strategy on the risk of recurrence and survival of breast cancer patients will ultimately show if hospitals responded adequately to the pandemic. In addition, this might provide valuable insights in how treatment can be improved.

## Supplementary Information

Below is the link to the electronic supplementary material.

Supplementary file1 (DOCX 57 KB)

## Data Availability

All data collected for the study, including a data dictionary defining each field in the set, will be made available via the NCR upon request and after approval of a study proposal from the date of publication. The plan for the statistical analysis will be made available by the corresponding author upon request.
