# Supplementary material for: Changes in breast cancer treatment during the COVID-19 pandemic: a Dutch population-based study
Source: Breast Cancer Res Treat. 2022 Nov 5;197(1):161–75. doi: 10.1007/s10549-022-06732-y (PMC9638417; doi:10.1007/s10549-022-06732-y)
Supplement: Supplementary file 1 — Supplementary file1 (DOCX 57 KB) [file 10549_2022_6732_MOESM1_ESM.docx]

# Changes in breast cancer treatment during the COVID-19 pandemic: a Dutch population-based study

Breast Cancer Research and Treatment

Anouk H Eijkelboom, Linda de Munck, C Willemien Menke-van der Houven van Oordt, Mireille J M Broeders, Desiree H J G van den Bongard, Luc J A Strobbe, Marc A M Mureau, Marc B I Lobbes, Pieter J Westenend, Linetta B Koppert, Agnes Jager, Ester J M Siemerink, Jelle Wesseling, Helena M Verkooijen, Marie-Jeanne T F D Vrancken Peeters, Marjolein L Smidt, Vivianne C G Tjan-Heijnen, Sabine Siesling, on behalf of the NABON-COVID-19 consortium and the COVID and Cancer Care-NL consortium

Address of correspondence:

Prof Dr Sabine Siesling, clinical epidemiologist

Department of Health Technology and Services Research

University of Twente

Drienerlolaan 5, 7522 NB, Enschede

the Netherlands

s.siesling@utwente.nl

+31 6 13 19 38 06

Supplementary Table 1 Logistic regression calculating the odds ratios and 95% confidence interval of the association between period of diagnosis and likelihood of receiving a specific treatment for patients with a non-screen-detected tumor, stratified by stage (sensitivity analysis group 1)

|  | Pre-COVID | Transition | Lockdown | Care restart |
| --- | --- | --- | --- | --- |
| DCIS, grade I-II |  |  |  |  |
| Surgery | 0.50 (0.26-0.94) | 0.22 (0.09-0.53) | 0.87 (0.18-4.34) | 0.36 (0.18-0.72) |
| DCIS, grade III |  |  |  |  |
| Surgery | 0.88 (0.18-4.21) | NA | NA | NA |
| Stage I |  |  |  |  |
| Neo-adjuvant chemotherapy | 1.56 (1.02-2.38) | 0.38 (0.16-0.91) | 1.91 (1.03-3.54) | 1.50 (1.02-2.21) |
| Neo-adjuvant endocrine therapy^a^ | 1.69 (0.89-3.22) | 2.83 (1.21-6.65) | 3.26 (1.54-6.90) | 0.78 (0.34-1.81) |
| Neo-adjuvant targeted therapy^b^ | 2.70 (1.27-5.74) | 0.80 (0.15-4.23) | 2.98 (1.01-8.82) | 1.33 (0.73-2.44) |
| Mastectomy | 0.93 (0.73-1.19) | 0.97 (0.65-1.42) | 0.94 (0.65-1.36) | 0.96 (0.77-1.21) |
| Adjuvant chemotherapy | 0.80 (0.57-1.12) | 1.75 (1.11-2.77) | 0.80 (0.48-1.33) | 0.89 (0.65-1.20) |
| Adjuvant endocrine therapy^a^ | 0.97 (0.77-1.21) | 1.09 (0.75-1.57) | 1.22 (0.86-1.71) | 1.05 (0.85-1.30) |
| Adjuvant targeted therapy^b^ | 1.95 (0.67-5.72) | 4.75 (0.25-88.65) | 2.20 (0.41-11.78) | 0.85 (0.40-1.77) |
| Adjuvant radiotherapy | 1.01 (0.81-1.25) | 0.95 (0.66-1.35) | 1.34 (0.94-1.93) | 1.05 (0.85-1.30) |
| IBR with autologous tissue^c^ | 0.73 (0.22-2.41) | NA | NA | 2.89 (1.50-5.55) |
| IBR with implant^c^ | 0.80 (0.50-1.28) | 1.47 (0.68-3.20) | 0.85 (0.41-1.77) | 1.21 (0.78-1.88) |
| IBR with autologous tissue and implant^c^ | 0.55 (0.07-4.11) | NA | NA | 0.47 (0.06-3.55) |
| Chemotherapy after radiotherapy^d^ | 1.37 (0.58-3.24) | 1.60 (0.45-5.65) | 0.60 (0.17-2.06) | 0.68 (0.36-1.31) |
| Stage II |  |  |  |  |
| Neo-adjuvant chemotherapy | 0.94 (0.75-1.17) | 0.73 (0.51-1.05) | 0.94 (0.66-1.34) | 1.22 (0.98-1.51) |
| Neo-adjuvant endocrine therapy^a^ | 1.18 (0.86-1.61) | 3.19 (2.22-4.58) | 1.43 (0.90-2.26) | 1.38 (1.04-1.84) |
| Neo-adjuvant targeted therapy^b^ | 1.25 (0.70-2.25) | 1.30 (0.50-3.38) | 1.93 (0.75-4.99) | 2.64 (1.37-5.07) |
| Mastectomy | 1.05 (0.88-1.25) | 0.92 (0.69-1.21) | 0.89 (0.67-1.18) | 1.12 (0.94-1.32) |
| Adjuvant chemotherapy | 1.05 (0.85-1.30) | 1.60 (1.17-2.18) | 1.16 (0.84-1.61) | 0.94 (0.76-1.16) |
| Adjuvant endocrine therapy^a^ | 1.02 (0.77-1.36) | 1.06 (0.68-1.65) | 0.76 (0.51-1.14) | 1.09 (0.82-1.43) |
| Adjuvant targeted therapy^b^ | 1.27 (0.56-2.92) | 5.10 (0.54-48.54) | 1.34 (0.42-4.28) | 1.81 (0.81-4.03) |
| Adjuvant radiotherapy | 0.92 (0.76-1.12) | 1.12 (0.82-1.54) | 1.07 (0.79-1.47) | 0.91 (0.76-1.10) |
| IBR with autologous tissue^c^ | 1.18 (0.56-2.49) | 1.27 (0.39-4.17) | 0.83 (0.20-3.45) | 2.00 (1.15-3.49) |
| IBR with implant^c^ | 0.96 (0.68-1.36) | 0.66 (0.35-1.24) | 0.45 (0.23-0.88) | 1.20 (0.88-1.63) |
| IBR with autologous tissue and implant^c^ | NA | 0.97 (0.13-7.19) | 2.92 (0.87-9.77) | 0.79 (0.24-2.59) |
| Chemotherapy after radiotherapy^d^ | 1.85 (1.11-3.07) | 1.63 (0.82-3.23) | 1.37 (0.66-2.87) | 1.00 (0.65-1.56) |
| Stage III |  |  |  |  |
| Neo-adjuvant chemotherapy | 0.67 (0.44-1.00) | 0.69 (0.39-1.23) | 1.63 (0.80-3.29) | 1.30 (0.83-2.03) |
| Neo-adjuvant endocrine therapy^a^ | 1.10 (0.64-1.87) | 2.27 (1.20-4.31) | 2.47 (1.27-4.80) | 0.80 (0.43-1.49) |
| Neo-adjuvant targeted therapy^b^ | 0.38 (0.15-0.99) | 1.00 (0.27-3.78) | 8.37 (0.96-73.04) | 0.93 (0.27-3.17) |
| Mastectomy | 0.78 (0.56-1.10) | 1.89 (1.05-3.42) | 0.90 (0.55-1.48) | 1.10 (0.77-1.58) |
| Adjuvant chemotherapy | 1.13 (0.77-1.66) | 1.26 (0.73-2.19) | 0.83 (0.46-1.51) | 1.46 (1.02-2.10) |
| Adjuvant endocrine therapy^a^ | 0.53 (0.31-0.90) | 1.31 (0.46-3.72) | 0.49 (0.23-1.05) | 2.04 (0.81-5.11) |
| Adjuvant targeted therapy^b^ | 0.95 (0.27-3.40) | 2.85 (0.54-15.00) | 2.15 (0.41-11.25) | 0.87 (0.22-3.45) |
| Adjuvant radiotherapy | 1.11 (0.63-1.96) | 0.77 (0.38-1.56) | 0.86 (0.40-1.85) | 0.89 (0.51-1.57) |
| IBR with autologous tissue^c^ | 2.08 (0.60-7.24) | NA | 2.83 (0.63-12.78) | 2.25 (0.74-6.83) |
| IBR with implant^c^ | 0.59 (0.27-1.32) | 0.84 (0.32-2.18) | 1.47 (0.66-3.27) | 1.45 (0.84-2.51) |
| IBR with autologous tissue and implant^c^ | 2.25 (0.26-19.19) | NA | NA | 2.05 (0.24-17.45) |
| Chemotherapy after radiotherapy^d^ | 2.95 (1.34-6.49) | 0.97 (0.36-2.57) | 3.50 (0.88-13.84) | 1.30 (0.67-2.52) |
| Stage IV |  |  |  |  |
| Neo-adjuvant chemotherapy | 0.95 (0.21-4.33) | 0.66 (0.19-2.32) | 2.30 (0.27-19.82) | 1.45 (0.34-6.28) |
| Neo-adjuvant endocrine therapy^a^ | 1.55 (0.30-7.93) | 2.54 (0.78-8.25) | NA | 0.83 (0.17-4.01) |
| Neo-adjuvant targeted therapy^b^ | NA | NA | NA | NA |
| Mastectomy | 0.84 (0.32-2.23) | 1.00 (0.35-2.88) | 1.34 (0.33-5.34) | 1.48 (0.58-3.81) |
| Adjuvant chemotherapy | 0.48 (0.10-2.36) | 0.35 (0.04-2.85) | 0.80 (0.15-4.16) | 2.77 (1.05-7.31) |
| Adjuvant endocrine therapy^a^ | 0.37 (0.07-1.98) | 1.27 (0.15-10.37) | NA | 1.15 (0.14-9.65) |
| Adjuvant targeted therapy^b^ | NA | NA | NA | 0.33 (0.03-4.22) |
| Adjuvant radiotherapy | 1.87 (0.51-6.86) | 1.22 (0.36-4.15) | 1.22 (0.25-6.03) | 1.42 (0.45-4.54) |
| IBR with autologous tissue^c^ | NA | NA | NA | NA |
| IBR with implant^c^ | NA | 3.48 (0.58-21.04) | NA | 2.28 (0.54-9.61) |
| IBR with autologous tissue and implant^c^ | NA | NA | NA | NA |
| Chemotherapy after radiotherapy^d^ | NA | NA | NA | 4.29 (0.43-42.44) |
| Reference: 2018/2019; Pre-COVID: weeks 1-8, 2020; Transition: weeks 9-12, 2020; Lockdown: weeks 13-17, 2020; Care restart: weeks 18-26, 2020  HER2: Human epidermal growth receptor 2, HR: Hormone receptor, IBR: Immediate breast reconstruction, NA: Too few patients for the analysis. Adjusted for age (<50, 50-74, >74) and tumor subtype (HR+/HER2+, HR+/HER2-, HR-/HER2+, HR-/HER2-).   1. These analyses only included patients with an HR+ tumor 2. These analyses only included patients with an HER2+ tumor 3. These analyses only included patients treated with a mastectomy 4. These analyses only included patients receiving adjuvant chemotherapy and radiotherapy. The likelihood of receiving chemotherapy after radiotherapy was compared with having radiotherapy after chemotherapy. | | | | |

Supplementary Table 2 Logistic regression calculating the odds ratios and 95% confidence interval of the association between period of diagnosis and likelihood of receiving a specific treatment for patients with a non-screen-detected tumor, stratified by tumor subtype (sensitivity analysis group 1).

|  | Pre-COVID | Transition | Lockdown | Care restart |
| --- | --- | --- | --- | --- |
| HR+/HER2+ |  |  |  |  |
| Neo-adjuvant chemotherapy | 1.25 (0.76-2.07) | 1.47 (0.63-3.47) | 3.40 (1.45-7.97) | 1.92 (1.17-3.15) |
| Neo-adjuvant endocrine therapy^a^ | 0.84 (0.33-2.13) | 2.13 (0.79-5.69) | 3.19 (1.41-7.19) | 1.00 (0.42-2.35) |
| Neo-adjuvant targeted therapy^b^ | 1.02 (0.63-1.65) | 1.50 (0.65-3.48) | 3.23 (1.42-7.34) | 1.82 (1.13-2.93) |
| Mastectomy | 0.78 (0.52-1.16) | 1.27 (0.67-2.39) | 0.78 (0.43-1.42) | 1.00 (0.69-1.45) |
| Adjuvant chemotherapy | 0.93 (0.57-1.50) | 1.17 (0.54-2.55) | 0.65 (0.29-1.43) | 0.82 (0.52-1.29) |
| Adjuvant endocrine therapy^a^ | 0.61 (0.40-0.95) | 2.15 (0.75-6.16) | 1.43 (0.65-3.12) | 1.05 (0.66-1.67) |
| Adjuvant targeted therapy^b^ | 1.13 (0.60-2.12) | 4.64 (1.14-18.91) | 2.08 (0.77-5.56) | 1.34 (0.72-2.50) |
| Adjuvant radiotherapy | 1.24 (0.77-1.98) | 0.67 (0.34-1.32) | 1.42 (0.69-2.94) | 1.00 (0.66-1.52) |
| IBR with autologous tissue^c^ | NA | 1.34 (0.17-10.90) | NA | 1.50 (0.42-5.30) |
| IBR with implant^c^ | 1.39 (0.63-3.04) | 1.37 (0.46-4.06) | 1.76 (0.58-5.32) | 1.40 (0.71-2.73) |
| IBR with autologous tissue and implant^c^ | 2.63 (0.30-23.00) | NA | 7.82 (0.82-74.68) | NA |
| Chemotherapy after radiotherapy^d^ | 0.54 (0.20-1.44) | 0.46 (0.06-3.28) | NA | 0.78 (0.3-2.02) |
| HR+/HER2- |  |  |  |  |
| Neo-adjuvant chemotherapy | 0.77 (0.61-0.97) | 0.66 (0.46-0.94) | 0.97 (0.69-1.38) | 1.04 (0.84-1.28) |
| Neo-adjuvant endocrine therapy^a^ | 1.28 (0.99-1.65) | 2.99 (2.21-4.03) | 1.62 (1.12-2.34) | 1.19 (0.93-1.54) |
| Neo-adjuvant targeted therapy^b^ | NA | NA | NA | NA |
| Mastectomy | 1.00 (0.85-1.17) | 1.10 (0.86-1.39) | 1.01 (0.78-1.29) | 1.13 (0.98-1.31) |
| Adjuvant chemotherapy | 1.01 (0.83-1.24) | 1.28 (0.95-1.73) | 1.03 (0.75-1.41) | 1.03 (0.85-1.25) |
| Adjuvant endocrine therapy^a^ | 1.00 (0.83-1.19) | 1.03 (0.78-1.37) | 0.90 (0.68-1.19) | 1.10 (0.92-1.31) |
| Adjuvant targeted therapy^b^ | NA | NA | NA | NA |
| Adjuvant radiotherapy | 0.98 (0.83-1.15) | 1.06 (0.81-1.38) | 1.00 (0.77-1.30) | 0.91 (0.78-1.06) |
| IBR with autologous tissue^c^ | 1.34 (0.73-2.47) | 0.53 (0.13-2.20) | 0.83 (0.26-2.67) | 2.44 (1.55-3.83) |
| IBR with implant^c^ | 0.82 (0.60-1.11) | 1.00 (0.62-1.61) | 0.65 (0.39-1.08) | 1.26 (0.96-1.64) |
| IBR with autologous tissue and implant^c^ | 0.23 (0.03-1.66) | 0.61 (0.08-4.48) | 0.63 (0.09-4.58) | 0.99 (0.39-2.49) |
| Chemotherapy after radiotherapy^d^ | 2.11 (1.32-3.39) | 1.33 (0.71-2.49) | 1.22 (0.62-2.39) | 1.09 (0.73-1.62) |
| HR-/HER2+ |  |  |  |  |
| Neo-adjuvant chemotherapy | 4.53 (1.44-14.32) | 0.61 (0.18-2.08) | 2.11 (0.64-6.98) | 1.43 (0.68-3.00) |
| Neo-adjuvant endocrine therapy^a^ | NA | NA | NA | NA |
| Neo-adjuvant targeted therapy^b^ | 4.74 (1.51-14.87) | 0.64 (0.19-2.16) | 2.20 (0.67-7.27) | 1.51 (0.72-3.15) |
| Mastectomy | 0.78 (0.42-1.48) | 1.07 (0.41-2.79) | 0.82 (0.37-1.82) | 1.13 (0.66-1.91) |
| Adjuvant chemotherapy | 0.34 (0.11-1.04) | 1.67 (0.51-5.48) | 0.23 (0.05-1.08) | 0.67 (0.32-1.44) |
| Adjuvant endocrine therapy^a^ | NA | NA | NA | NA |
| Adjuvant targeted therapy^b^ | 3.18 (0.79-12.71) | 3.19 (0.34-30.09) | 1.13 (0.28-4.59) | 0.74 (0.33-1.69) |
| Adjuvant radiotherapy | 2.03 (0.88-4.67) | 1.08 (0.33-3.51) | 0.93 (0.37-2.35) | 1.24 (0.67-2.29) |
| IBR with autologous tissue^c^ | 2.15 (0.23-20.42) | NA | 2.33 (0.23-23.04) | 0.85 (0.10-7.53) |
| IBR with implant^c^ | 0.21 (0.03-1.70) | 0.79 (0.08-7.39) | 0.52 (0.10-2.71) | 0.71 (0.24-2.06) |
| IBR with autologous tissue and implant^c^ | NA | NA | NA | NA |
| Chemotherapy after radiotherapy^d^ | NA | 1.18 (0.17-8.12) | NA | 0.31 (0.04-2.10) |
| HR-/HER2- |  |  |  |  |
| Neo-adjuvant chemotherapy | 1.30 (0.83-2.02) | 0.46 (0.25-0.85) | 0.97 (0.50-1.89) | 2.33 (1.42-3.84) |
| Neo-adjuvant endocrine therapy^a^ | NA | NA | NA | NA |
| Neo-adjuvant targeted therapy^b^ | NA | NA | NA | NA |
| Mastectomy | 1.06 (0.77-1.47) | 0.71 (0.43-1.19) | 0.74 (0.43-1.25) | 0.79 (0.56-1.11) |
| Adjuvant chemotherapy | 1.10 (0.79-1.53) | 2.86 (1.71-4.80) | 1.35 (0.81-2.22) | 1.25 (0.90-1.75) |
| Adjuvant endocrine therapy^a^ | NA | NA | NA | NA |
| Adjuvant targeted therapy^b^ | NA | NA | NA | NA |
| Adjuvant radiotherapy | 0.73 (0.52-1.03) | 1.06 (0.61-1.86) | 2.84 (1.27-6.35) | 1.45 (0.96-2.20) |
| IBR with autologous tissue^c^ | 0.62 (0.08-4.79) | NA | NA | 3.25 (1.04-10.11) |
| IBR with implant^c^ | 0.91 (0.47-1.76) | 0.34 (0.07-1.67) | 0.54 (0.15-1.98) | 1.31 (0.68-2.53) |
| IBR with autologous tissue and implant^c^ | NA | NA | 7.33 (0.78-68.86) | NA |
| Chemotherapy after radiotherapy^d^ | 5.53 (1.32-23.17) | 2.31 (0.69-7.73) | 1.12 (0.37-3.42) | 1.16 (0.57-2.38) |
| Reference: 2018/2019; Pre-COVID: weeks 1-8, 2020; Transition: weeks 9-12, 2020; Lockdown: weeks 13-17, 2020; Care restart: weeks 18-26, 2020  HER2: Human epidermal growth receptor 2, HR: Hormone receptor, IBR: Immediate breast reconstruction, NA: Not applicable or too few patients for the analysis. Adjusted for age (<50, 50-74, >74) and stage.   1. These analyses only included patients with an HR+ tumor 2. These analyses only included patients with an HER2+ tumor 3. These analyses only included patients treated with a mastectomy 4. These analyses only included patients receiving adjuvant chemotherapy and radiotherapy. The likelihood of receiving chemotherapy after radiotherapy was compared with having radiotherapy after chemotherapy. | | | | |
